# Evaluation of the Xpert MTB/RIF test for the diagnosis of childhood pulmonary tuberculosis in Uganda: a cross-sectional diagnostic study

**DOI:** 10.1186/1471-2334-13-133

**Published:** 2013-03-12

**Authors:** Moorine Penninah Sekadde, Eric Wobudeya, Moses L Joloba, Willy Ssengooba, Harriet Kisembo, Sabrina Bakeera-Kitaka, Philippa Musoke

**Affiliations:** 1 Department of Paediatrics and Child Health, School of Medicine, College of Health Sciences, Makerere University, Kampala, Uganda; 2 Directorate of Paediatrics and Child Health, Mulago National Referral Hospital, Kampala, Uganda; 3 Makerere University-John Hopkins University (MU-JHU) Research Collaboration, Kampala, Uganda; 4 Department of Microbiology, School of Medicine, College of Health Sciences, Makerere University, Kampala, Uganda; 5 Department of Radiology, Mulago National Referral Hospital, Kampala, Uganda

**Keywords:** Children, Pulmonary tuberculosis, Sensitivity, Specificity, Xpert MTB/RIF

## Abstract

**Background:**

The diagnosis of childhood tuberculosis remains a challenge worldwide. The Xpert MTB/RIF test, a rapid mycobacteria tuberculosis diagnostic tool, was recommended for use in children based on data from adult studies. We evaluated the performance of the Xpert MTB/RIF test for the diagnosis of childhood pulmonary tuberculosis using one induced sputum sample and described clinical characteristics associated with a positive Xpert MTB/RIF test. The sputum culture on both Lowenstein-Jensen (LJ) and Mycobacteria Growth Indicator Tube (MGIT) was the gold standard.

**Methods:**

We consecutively enrolled 250 Ugandan children aged 2 months to 12 years with suspected pulmonary tuberculosis between January 2011 and January 2012 into a cross-sectional diagnostic study at a tertiary care facility in Uganda.

**Results:**

We excluded data from 15 children (13 contaminated culture and 2 indeterminate MTB/RIF test results) and analysed 235 records. The Xpert MTB/RIF test had a sensitivity of 79.4% (95% CI 63.2 - 89.7) and a specificity of 96.5% (95% CI 93 – 98.3). The Xpert MTB/RIF test identified 13 of the 14 (92.9%) smear positive-culture positive and 14 of the 20 (70%) smear negative -culture positive cases. The Xpert MTB/RIF identified twice as many cases as the smear microscopy (79.4% Vs 41.2%). Age > 5 years (OR 3.3, 95% CI 1.4 – 7.4, p value 0.005), a history of Tuberculosis (TB) contact (OR 2.4, 95% CI 1.1 – 5.2, p value 0.03), and a positive tuberculin skin test (OR 4.1, 95% CI 1.7 – 10, p value 0.02) was associated with a positive Xpert MTB/RIF test. The median time to TB detection was 49.5 days (IQR 38.4-61.2) for LJ, and 6 days (IQR 5 – 11.5) for MGIT culture and 2 hours for the Xpert MTB/RIF test.

**Conclusion:**

The Xpert MTB/RIF test on one sputum sample rapidly and correctly identified the majority of children with culture confirmed pulmonary tuberculosis with high specificity.

## Background

Diagnosis of childhood pulmonary tuberculosis (PTB) has inherent challenges such as the paucibacillary nature of the disease, difficulties in obtaining sufficient sputum samples, intrinsic limitations of available tests, and overlap of respiratory clinical presentation in Human Immune Deficiency Virus (HIV) infection [1-3]. The current methods used in the diagnosis of tuberculosis (TB) have not greatly impacted on the diagnostic process in the paediatric population and this has resulted into delayed diagnosis or misdiagnosis and subsequent over or under treatment [1,3,4]. Even though sputum culture is considered the gold standard diagnostic tool for PTB, it is only able to detect approximately 30 – 40% of cases with probable tuberculosis in children [4-6]. While smear microscopy is inexpensive, simple to perform, and has a quick turnaround time, the proportion of positive samples among children is minimal due to the low bacillary burden observed in childhood tuberculosis [4]. Newer tools such as the Nucleic Acid Amplification Tests have a slightly higher sensitivity of 40 – 60% [4]. Other diagnostic tests including the Tuberculin Skin Test (TST) and the Interferon γ Release Assays are limited in differentiating between latent infection and active disease [4]. When compared to adults, children have a higher risk of severe progressive disease and death due to tuberculosis hence the urgent need for a rapid and accurate diagnostic tool [2].

The recent innovation of the Xpert MTB/RIF test by Cepheid, Sunnyvale, CA (USA) has greatly transformed the field of TB diagnostics [7]. The Xpert MTB/RIF test simultaneously detects mycobacteria tuberculosis (MTB) and resistance to rifampicin using real-time polymerase chain reaction (PCR) analysis and avails results within 2 hours [7]. The other main advantage of the XpertMTB/RIF compared to the traditional PCR methods is the possibility to use it in a relatively peripheral laboratory setting because it is fully automated.

In the policy statement on the Xpert MTB/RIF test in 2011, the World Health Organisation (WHO) recommended the use of the test as an initial diagnostic tool among children with suspected HIV associated TB or Multi-Drug Resistant TB based on successful data among adults [8-10]. Boehme et al. documented sensitivity of 98.2% and 72.5% among adults with culture positive-smear positive and culture positive–negative culture positive tuberculosis respectively in a multicenter study conducted in Peru, Azerbaijan, South Africa, and India [9]. There are limited published data on the utility of the Xpert MTB/RIF test in the paediatric population with tuberculosis [11,12]. A recent study by Nicol MP et al. found an overall sensitivity of 100% for smear positive-culture positive cases, 61.1% for smear negative- culture positive, and specificity of 98.8% when two induced sputum samples was assessed among children aged less than 15 years with suspected pulmonary tuberculosis in Cape Town, South Africa [11]. Similar findings were reported by Rachow et al. in a prospective study conducted in Tanzania where the Xpert MTB/RIF test detected 100% of smear positive-culture positive cases and 66.6% for smear negative-culture positive cases [12]. We therefore aimed to evaluate the performance of the Xpert MTB/RIF test for the diagnosis of childhood pulmonary tuberculosis in Uganda and describe clinical characteristics associated with a positive Xpert MTB/RIF test using one induced sputum sample and sputum culture on both Lowenstein-Jensen (LJ) and Mycobacteria Growth Indicator Tube (MGIT) as the gold standard.

## Methods

### Study participants

We conducted a cross-sectional diagnostic study at the Mulago National Referral Hospital in Kampala, Uganda on both paediatric in-patients and out-patients aged 2 months to 12 years with suspected pulmonary tuberculosis between January 2011 and January 2012. Eligibility for enrolment was based on the WHO case definition for a TB suspect [13,14] and included having a persistent cough of 2 weeks or more and one of the following: household TB contact, unexplained weight loss or failure to gain weight, and unexplained fever for 2 weeks or more. We excluded children who were on anti- tuberculous therapy and those in whom sputum could not be obtained or sputum induction was contraindicated. The contraindications to sputum induction were adapted from the WHO guidelines for sputum induction and included: severe respiratory distress (including rapid breathing, wheezing, hypoxia); intubated patient; bleeding: low platelet count, bleeding tendency, severe nosebleeds (symptomatic or platelet count <50/ml blood); reduced level of consciousness and history of significant asthma (diagnosed and treated by a clinician) [13]. The exclusion criterion “children in whom sputum could not be obtained” was defined after systematic attempt of sputum induction.

### Study procedure

The study was approved by the School of Medicine Research and Ethics committee of Makerere University College of Health Sciences (# REC REF 2011–005). The children were enrolled upon provision of informed consent from the primary caregivers and assent for those older than 8 years. We conducted consecutive enrolment until the desired sample size was achieved. Upon enrolment, a clinical history and physical examination were conducted. The investigations performed included the TST, Complete Blood Count, HIV testing in children whose HIV status was unknown, and chest radiography. Each child’s nutritional status was assessed using the WHO child growth standards [15]. Weight for length/weight for height was used for children < 5 years while Body Mass Index (BMI) for Age was used for children ≥ 5 years. Severe wasting was defined as weight for length or weight for height or BMI for age Z score which was less than – 3SD.

### Chest radiography

The chest radiographs (Antero-Posterior views) were evaluated for pulmonary tuberculosis by two independent reviewers who used a standardised reporting form and were blinded to the results of the other investigations. A third independent reviewer evaluated a radiograph in the event that there was discordant reporting between the two reviewers. The reviewers classified the chest radiographs into probable TB (findings consistent with TB), possible TB (atypical findings), and TB unlikely (findings not consistent with TB or normal chest radiograph) based on the radiological features present [16]. Findings consistent with TB included lymphadenopathy for children 5 years and below while, a typical adult type of chest radiograph appearance with predominance of upper lobe infiltrates with or without cavitation was used for older children. A miliary pattern was categorized as probable TB.

### Tuberculin skin test

A qualified trained study nurse administered 0.1 ml of purified protein derivative (PPD; 2TU, PPD RT23, Staten Serum Institute, Denmark, Copenhagen) intradermally on the volar aspect of the less dominant forearm. The induration at the site of administration was measured in millimetres after 48–72 hours of administration. A positive skin test was defined as > 5 mm of the transverse induration in children with HIV infection and > 10 mm in children without HIV infection.

### Sputum sample collection

A qualified trained study nurse performed sputum induction using nebulised 3% hypertonic saline after a minimum of 3 hours fast. The study participants underwent premedication with nebulised salbutamol prior to the administration of nebulised 3% hypertonic saline. A maximum of two attempts were conducted to obtain an adequate sample of at least 3 mls of sputum. However whatever volume of samples obtained was sent for analysis. The study participants had pulse oximetry monitoring during and for one hour after the procedure. The sputum sample was collected in a sterile container which was placed on an ice pack in a cool box, taken to an accredited mycobacteriology laboratory (Makerere University Department of Microbiology – Mycobacteriology Laboratory) and processed within two hours of collection. Digestion-Decontamination of sputum by N- acetyl L- cysteine (NALC)-Sodium Hydroxide method was performed prior to making smears for fluorochrome staining with Auramine O Phenol and examined according to standards. Samples were inoculated into the Mycobacteria Growth Indicator Tube (MGIT) (Becton Dickson, Franklin Lakes, NJ) and Lowenstein-Jensen (LJ) slants and incubated for 6 weeks on MGIT and 8 weeks on LJ culture. At least 0.5 ml of the sediment sample was stored at – 20°C for the Xpert MTB/RIF test. Due to the delay in delivery of the Xpert MTB/RIF cartridges and sample reagents, we were unable to run the Xpert MTB/RIF test on the freshly collected sputum samples. A culture was considered positive if mycobacterium tuberculosis growth was confirmed on either LJ or MGIT media. A culture was considered negative if no growth was confirmed on both LJ and MGIT media, if one culture result was negative and the other was contaminated. A culture was considered contaminated if both LJ and MGIT demonstrated contamination. Mycobacteria Other Than Tuberculosis (MOTT) was considered if both LJ and MGIT culture media grew MOTT. Mycobacterial identification was done using Capilia TB Neo™ (TAUN, Numazu, Japan) assay. The frozen samples were thawed to room temperature prior to addition of the Xpert MTB/RIF sample reagent. Three parts (1.5 ml) of the sample reagent were added to one part (0.5 ml) of the sediment sample as per the manufacturer’s guide. The mixture was shaken and incubated at room temperature for 15 minutes. The mixture was then transferred to a pre labeled cartridge and subsequently loaded into the Xpert MTB/RIF machine. The results were printed out after a 2 hours cycle was complete. An Xpert MTB/RIF test was considered positive if MTB was detected and negative if MTB was not detected. We were unable to run a repeat test for the indeterminate MTB/RIF test result as the stored sputum sample was insufficient. Drug susceptibility testing was not performed in children identified with rifampicin resistance based on Xpert test due to cost limitations. The indeterminate test results were reported as such and were excluded from analysis. Quality control measures for the Xpert MTB/RIF test included negative and positive control tests for every new batch of Xpert MTB/RIF cartridges; monthly negative and positive control tests; monthly room swabs for mycobacteria tuberculosis contamination; and automated quality control for PCR. The laboratory technologists processing the sputum samples for culture and Xpert MTB/RIF were blinded to the results of the other test.

### Blood sample collection

Using aseptic technique, 3mls of venous blood were drawn for CBC and HIV testing under aseptic conditions. The CBC assays were performed using Beckman Dickinson Coulter Counter (5 Part-Differential). HIV rapid testing was conducted on all children without a documented HIV status. HIV exposed infants aged 18 months or younger had an HIV DNA PCR done to confirm the diagnosis. The HIV rapid test algorithm used was that recommended by the Ministry of Health in Uganda.

### Statistical considerations

#### Sample size

At the time we started data collection, there were no published data on evaluation of the Xpert MTB/RIF among children. Sensitivity and specificity normograms with a fixed false positive rate of 5% were used to calculate the sample size for this study [17]. Using a prevalence of culture confirmed pulmonary tuberculosis of 30% in a Ugandan paediatric population,[5] sensitivity of the Xpert MTB/RIF test in the adult population of 98.2% [9] and 95% confidence interval, we obtained a sample size 250 study participants.

#### Data analysis

The data were captured using EPI INFO version 3.5.3, analysed using STATA version 10 and OpenEpi. We described data for all enrolled study participants but excluded data of children with indeterminate MTB/RIF test results and contaminated sputum cultures from the diagnostic accuracy analysis. Study population characteristics were described using proportions with 95% CI, means with standard deviations, and medians with interquartile ranges (IQR). We excluded data of children with indeterminate MTB/RIF test results and contaminated sputum culture results from the diagnostic accuracy analysis. Sensitivity, specificity, positive and negative predictive values, positive and negative likelihood ratios with their 95% CI for the Xpert MTB/RIF test were calculated using sputum culture as the gold standard. The chi square statistical test was used to compare the proportions. We explored the clinical characteristics associated with a positive Xpert MTB/RIF test result using multivariate logistic regression analysis. The clinical characteristics with a p value of < 0.2 on bivariate analysis and those associated with PTB were entered into the logistic regression model using the backward stepwise method. P values less than 0.05 were considered statistically significant.

## Results

### Descriptive statistics

**Figure 1:**
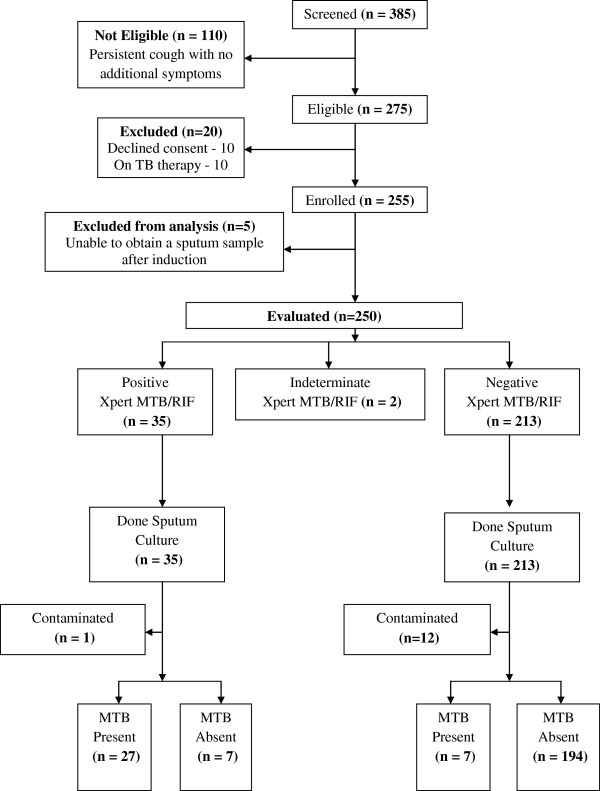
Study profile.

We enrolled 255 (92.7%) of 275 eligible study participants and described data for 250 study participants. (See Figure 1) All the 250 study participants had sputum cultures (both LJ and MGIT) and Xpert MTB/RIF tests conducted. The median age was 36 months (IQR 16 – 74.5) and 41.6% were HIV positive. The study participant characteristics are as shown in Table 1.

**Table 1:** Characteristics of study participants with suspected pulmonary tuberculosis (N = 250)

**Characteristic**		**Frequency**	**Percentage**
**Age**	< 1 year	42	16.8
	1-5 years	131	52.4
	> 5 years	77	30.8
**Sex**	Male	134	53.6
	Female	116	46.4
**Patient status**	Inpatient	211	84.4
	Outpatient	39	15.6
**HIV status**	Positive	104	41.6
	Negative	146	58.4
**History of TB contact**	Yes	86	34.4
	No	164	65.6
**Prior TB diagnosis**	Yes	18	7.2
	No	232	92.8
**Previous pneumonia admission**	Yes	66	26.4
	No	184	73.6
**History of INH prophylaxis**	Yes	1	0.4
	No	249	99.6
**Tuberculin skin test**	Positive	76	30.4
	Negative	174	69.6
Severe wasting*	Yes	68	27.2
	No	182	72.8
**BCG scar**	Yes	169	67.6
	No	81	32.4

* Weight for height/length < 5 years, BMI/Age ≥ 5 years which was < -3SD.

Fifteen children (6%, 95% CI 3.4- 9.7) had a positive sputum smear for acid fast bacilli. There were thirty five children (14%, 95% CI 9.9 – 18.9) with a positive sputum culture for MTB and thirty five children (14%, 95% CI 9.9 – 18.9) with a positive Xpert MTB/RIF test. Two (5.7% 95% CI 0.6 – 19.1) of the 35 children with a positive MTB/RIF test had rifampicin resistance detected. We documented a positive Xpert MTB/RIF test in 14 of the 15 (93.3%, 95% CI 68.1 - 99.8) positive smears. Twelve (34.3%) of the children with a positive sputum culture were HIV positive while thirteen children (37.1%) with a positive Xpert MTB/RIF test were HIV positive. There was no statistically significant difference in the positivity rate for the Xpert MTB/RIF results between HIV positive and negative children. We documented a positive Xpert MTB/RIF test in 9.5% (4 of 42), 9.9% (13 of 131), and 23.4% (18 of 77) of children aged < 1 year, 1 – 5 years, and > 5 years respectively. Three children (3/35, 8.6%) had MTB growth on LJ media and MOTT on MGIT media. Two of the three children had a positive Xpert MTB/RIF test.

Thirteen sputum cultures (5.2%, 95% CI 2.8 – 8.7%) were classified as contaminated while 2 Xpert MTB/RIF test results (0.8%, 95% CI 0.1 – 2.9) were indeterminate. While the median time to detection was 49.5 days (IQR 38.4-61.2) for LJ and 6 days (IQR 5 – 11.5) for MGIT culture, it was 2 hours for the Xpert MTB/RIF test.

Chest radiographs for 235 study participants were reviewed and classified as probable TB (80/235, 34% (95% CI 28–40.5)); possible TB (96/235, 40.9% (95% CI 34.5-47.4)), and TB unlikely (46/235, 19.6% (95% CI 14.7-25.2)). Thirteen of 235 (5.5%, 95% CI 3–9.3) radiograph reviews were inconclusive in that all the three reviewers did not agree. Fifteen chest radiographs were not reviewed as these children were lost to follow up before the radiographs were taken.

### Diagnostic test results

For diagnostic accuracy, we excluded data from 15 children (13 contaminated culture and 2 indeterminate MTB/RIF test results) and analysed 235 records. The Xpert MTB/RIF test identified MTB in 27 of the 34 culture confirmed cases demonstrating a sensitivity of 79.4% (95% CI 63.2 – 89.7) and 7 of 201 culture negative cases, a specificity of 96.5% (95% CI 93 – 98.3). More diagnostic parameters are shown in Table 2. Fourteen of the 34 culture positives were smear positive (41.2%, 95% CI 24.6 – 59.3). The Xpert MTB/RIF test identified twice as many cases as the smear microscopy (79.4% Vs 41.2%). The Xpert MTB/RIF test identified 13 of 14 smear positive-culture positive (93%, 95% CI 66–99.8) and 14 of 20 smear negative -culture positive cases (70%, 95% CI 46–88). Twenty one of 221 (9.5%, 95% CI 6 – 14.2) negative smears were Xpert MTB/RIF positive. The Xpert MTB/RIF test had a sensitivity of 93.3% (95% CI 70.2 – 98.8) and specificity of 94.8% (95% CI 85.9 – 98.2) among children aged 5 years and above. The sensitivity and specificity of the Xpert MTB/RIF test was 68.2% (95% CI 46 – 84.6) and 97.2% (95% CI 93 – 98.9) respectively among children aged 5 years and below. The clinical characteristics which were independently associated with a positive Xpert MTB/RIF test included age > 5 years, a positive history of TB contact, and a positive tuberculin test (Table 3).

**Table 2:** The Xpert MTB/RIF test evaluation among children with suspected pulmonary TB (N = 235)

	**Overall (n = 235)**		**HIV positive (n = 99)**		**HIV negative (n = 136)**	
	**Estimate**	**95% CI**	**Estimate**	**95% CI**	**Estimate**	**95% CI**
**Sensitivity (%)**	27/34, 79.4	63.2 - 89.7	9/11,81.8	52.3-94.9	18/23, 78.3	58.1-90.3
**Specificity (%)**	194/201, 96.5	93- 98.3	84/88,95.5	88.9- 98.2	110/113, 97.4	92.5- 99.1
**PPV (%)**	79.4	63.2- 89.7	69.2	42.4- 87.3	85.7	65.4- 95
**NPV (%)**	96.2	93-98.3	97.7	91.9-99.4	95.7	90.2- 98.1
**Diagnostic accuracy (%)**	94	90.3- 96.4	93.9	87.4-97.2	94.1	88.8- 97
**Likelihood ratio (+)**	22.8	16.9 - 30.7	18	10.5 - 30.8	29.5	14.9- 58.4
**Likelihood ratio (-)**	0.2	0.2- 0.3	0.2	0.1-0.5	0.2	0.2 - 0.3

*PPV *– Positive Predictive Value; *NPV *– Negative Predictive Value.

**Table 3:** Clinical characteristics associated with a positive xpert MTB/RIF test (N = 235)

**Variable**	**Crude OR (95% CI)**	**P value**	**Adjusted OR (95% CI)**	**P value**
**Age (> 5 years Vs ≤ 5 years)**	2.6 (1.2 -5.4)	0.012	3.3 (1.4 - 7.4)	0.005
**Sex (female vs. male)**	1.1 (0.5 – 2.2)	0.9		
**Positive HIV status**	0.8 (0.4 - 1.7)	0.6	1.2 (0.5 – 3.0)	0.7
**Positive history of TB contact**	2.8 (1.4 - 5.9)	0.006	2.4 (1.1 – 5.2)	0.03
**Prior TB diagnosis**	0.8 (0.2 - 3.8)	0.8		
**Positive TST**	3.4 (1.6 - 7.3)	0.001	4.1 (1.7 - 10)	0.002
**BCG scar present**	0.7 (0.3 - 1.5)	0.4		

## Discussion

In the policy statement on the Xpert MTB/RIF test in 2011, the WHO recommended the use of the test as an initial diagnostic tool among children with suspected HIV associated TB or MDR TB based on significant data among adults [8].

The proportion of children (14%) with a positive Xpert MTB/RIF was similar to that reported (13%) from a larger and younger paediatric South African population in a high TB/HIV prevalent area [11]. The Xpert MTB/RIF test correctly identified 79.4% and 96.5% of children with and without culture confirmed pulmonary tuberculosis respectively. Using the Fagan normogram, at a childhood PTB prevalence of 14% from our study and an overall positive likelihood ratio of 23, the probability of pulmonary tuberculosis when the Xpert MTB/RIF test is positive is 80% while the probability of pulmonary tuberculosis when the Xpert MTB/RIF test is negative is 3% [18]. This gives the clinician sufficient confidence to make a diagnosis of pulmonary tuberculosis when the Xpert MTB/RIF test is positive. A negative Xpert MTB/RIF test does not exclude a diagnosis of pulmonary tuberculosis given the fact that the test was unable to identify 20.6% of children with culture confirmed pulmonary tuberculosis. A clinical decision in the context of the patient is therefore important in initiating anti-tuberculous therapy in a child who has a negative Xpert MTB/RIF test. Just as in the South African and Mbeya studies, our results demonstrate that the Xpert MTB/RIF test identified majority of the culture confirmed cases with high specificity [11,12]. While we did not perform a second Xpert MTB/RIF test, our results are highly comparable to the studies conducted in high TB prevalent areas in South Africa and Mbeya where multiple sputum samples were used [11,12]. We do not discourage the use of a second Xpert MTB/RIF test but the decision needs to be guided by the available resources. We also documented a lower sensitivity (70%) among smear negative-culture positive case and twice as many cases with the Xpert MTB/RIF test as did smear microscopy. The documentation of twice as many cases with the Xpert MTB/RIF test as with smear microscopy also underscores the clinical relevance of the Xpert MTB/RIF test. Studies conducted in the adult population have reported slightly higher sensitivity values of nearly 73% among smear negative culture positive cases [9,10]. The differences between adults and children could be related to the inherent low sensitivity of the culture method, higher AFB load in adults, and the occurrence of smear negative primary disease among children.

Although the Xpert MTB/RIF test was unable to identify nearly 21% of the culture positive cases, the median time to TB detection was significantly shorter than that for LJ and MGIT culture methods. The Xpert MTB/RIF test also presents a hands-free sample processing technique which can be operated by an individual with minimal training [7]. This greatly impacts on the time taken to process a sputum sample for smear microscopy and culture as well as limit a laboratory technician’s exposure to MTB. We documented higher contamination rates with the culture method compared to the indeterminate results observed with the Xpert MTB/RIF test. A feasibility and effectiveness study conducted among adults with suspected tuberculosis or multidrug-resistant tuberculosis at the District and Sub District health centers in TB endemic resource limited areas reported that the Xpert MTB/RIF test is effective in reducing the time to initiation of anti-tuberculous therapy as well as lost to follow up cases due to delays in making a diagnosis [19]. The Xpert MTB/RIF test may be more expensive than smear microscopy but the costs are similar to running sputum culture and drug susceptibility testing [19].

Unlike the South African study, both the Mulago and Mbeya studies found no statistical difference in the Xpert MTB/RIF positivity rate between HIV positive and negative children There was a larger proportion of HIV infected children in the Mulago (40.9%) and Mbeya (58.9%) studies when compared to the South African study (24%) [11,12]. Also, children with HIV commonly present with respiratory complications that may mimic pulmonary TB [2].

Due to the limited availability of the Xpert MTB/RIF test, we explored the clinical characteristics associated with a positive Xpert MTB/RIF test in order to help clinicians predict which child is likely to have a positive test. We found that age > 5 years, a positive TST, and a positive history of TB contact were independently associated with a positive Xpert MTB/RIF test. Unlike younger children who get paucibacillary primary disease, older children especially those above 10 years of age are more likely to get reactivation/cavitatory disease there by increasing the likelihood of a positive Xpert MTB/RIF test [20]. A history of TB exposure increases the likelihood of occurrence of TB in a child with suspected pulmonary tuberculosis. A positive TST response is a marker of TB exposure and also increases the likelihood of occurrence of TB in a child with suspected pulmonary tuberculosis. The other studies have mainly been descriptive and have not looked at factors associated with a positive Xpert MTB/RIF test.

We documented three children who had both MTB and MOTT LJ and MGIT media. Of these, two had a positive Xpert MTB/RIF test. This suggests the possibility for co infection with both MTB and MOTT. We reported two children with rifampicin resistance among those with a positive Xpert MTB/RIF test. It is possible that the resistance was secondary as opposed to primary because the two children had defaulted several times due to medication shortages. These children were referred to the National TB and Leprosy Program for further management and follow-up. Since we did not perform drug susceptibility testing, we were unable to comment on the performance of the Xpert MTB/RIF test in identifying rifampicin resistance.

This is the first study in Uganda and one of the very few large studies worldwide that have evaluated the performance of the Xpert MTB/RIF test in the diagnosis of pulmonary tuberculosis among children. We also explored the factors associated with a positive Xpert test which had not been described in earlier studies. The fact that all study participants were hospital cases might affect the generalisability of these study findings to the community setting. The low sensitivity of sputum culture for PTB in children makes it an inadequate diagnostic tool to compare with especially when more sensitive tools are being assessed. Due to the small amounts of the sputum sample stored, we were unable to run a repeat Xpert MTB/RIF test in cases where the initial test was indeterminate. Even though we performed the Xpert MTB/RIF test on frozen samples, an earlier study by Theron G et al. demonstrated no significant difference in sensitivity when the sputum samples were frozen [21].

Even though an earlier study conducted among adults demonstrated similar performance of the Xpert MTB/RIF test for untreated and treated sputum, [9] we recommend a similar study in the pediatric population which is faced with paucibacillary primary disease and difficulties in obtaining adequate sputum samples. We also suggest a study to assess the performance of the Xpert MTB/RIF test at the District and Sub-District levels as the population at a national referral hospital differs from that at the lower health facilities. A study at the lower health facilities will also assess the feasibility of performing sputum induction at these facilities. Uganda as a developing country is faced with challenges including costs of purchase and maintenance, infrastructure at the lower health facilities, and power shortages, thereby hindering the sustainability of this service. The WHO has however revised the purchase costs to cater for resource poor countries including Uganda. Also, efforts to improve on the diagnosis of pediatric TB can be used as a platform to argue for better government support and commitment in managing TB among children.

## Conclusions

The Xpert MTB/RIF test on one sputum sample rapidly and correctly identified the majority of children with culture confirmed pulmonary tuberculosis with high specificity. Age > 5 years, a positive TST, and a positive history of TB contact were positively associated with a positive Xpert MTB/RIF test.

## Competing interests

The authors declare that they have no competing interests.

## Authors’ contributions

MPS, PM, EW, and MLJ conceived the idea, obtained funding, and primarily drafted the article. MPS coordinated the data collection, analysis, and interpretation of the data under the supervision of PM, EW, and MLJ. WS coordinated the microbiological testing. SBK and HK reviewed the chest radiographs. All authors read and approved the final manuscript. PM is the study guarantor.

## Pre-publication history

The pre-publication history for this paper can be accessed here:

http://www.biomedcentral.com/1471-2334/13/133/prepub
